# Identification and analysis of risk factors for poor prognosis in patients with acute ischemic stroke undergoing thrombolytic therapy

**DOI:** 10.1097/MD.0000000000046575

**Published:** 2025-12-19

**Authors:** Cuihong Ma, Lixia Gao, Zengkun Hong, Jing Xu, Lei Chen

**Affiliations:** a Department of Neurology, Clinical College of Neurology, Neurosurgery, and Neurorehabilitation, Tianjin Medical University, Dagang, Binhai New Area, Tianjin, China; b Chengde Medical University, Shuangqiao District, Chengde City, Hebei Province, China; c Department of Neurology, Chengde Central Hospital, Shuangqiao District, Chengde City, Hebei Province, China.

**Keywords:** acute ischemic stroke, decision curve analysis, nomogram, predictive modeling, prognosis, risk factors, thrombolysis

## Abstract

Early identification of patients at high risk of poor prognosis after thrombolytic therapy for acute ischemic stroke (AIS) is essential for optimizing clinical management. This study aimed to develop and internally validate a prognostic nomogram integrating clinical and laboratory variables. This retrospective study included 286 AIS patients treated with recombinant tissue plasminogen activator between January 2022 and August 2024. Patients were categorized into favorable (modified Rankin Scale ≤ 2, n = 198) and poor prognosis groups (modified Rankin Scale > 2, n = 88) based on outcomes at 90 days post-treatment. Clinical data, including demographic information, comorbidities, National Institutes of Health Stroke Scale (NIHSS) scores, and laboratory parameters (e.g., white blood cell [WBC] count, neutrophil count, and D-dimer levels), were collected. Multivariate logistic regression analysis identified independent predictors of poor prognosis. A nomogram was developed to predict prognosis, with performance evaluated through receiver operating characteristic analysis, Bootstrap resampling (n = 1000), and calibration curves. Clinical utility was assessed using decision curve analysis. Multivariate analysis identified 5 independent predictors of poor prognosis: diabetes (odds ratio [OR] = 6.511, 95% confidence interval [CI]: 1.667–24.605, *P* = .008), admission NIHSS score (OR = 1.354, 95% CI: 1.097–1.565, *P* = .001), WBC count (OR = 1.459, 95% CI: 1.114–1.786, *P* = .002), neutrophil count (OR = 1.402, 95% CI: 1.037–1.758, *P* = .022), and D-dimer level (OR = 2.088, 95% CI: 1.360–2.988, *P* = .001). The nomogram showed excellent discrimination (AUC = 0.896), good calibration (Hosmer–Lemeshow, *P* = .856), and clinical utility. Internal validation yielded a concordance index of 0.728. This retrospective study suggests that diabetes, NIHSS score, WBC count, neutrophil count, and D-dimer levels may be useful predictors of poor prognosis in thrombolysed AIS patients. A nomogram based on these factors showed good discriminative ability and clinical utility. Further prospective, multicenter validation is needed to confirm its applicability in routine practice.

## 1. Introduction

Acute ischemic stroke (AIS) is a leading cause of morbidity and mortality worldwide, and its management remains a critical challenge in clinical neurology. Thrombolytic therapy, particularly intravenous recombinant tissue plasminogen activator (rt-PA), has become the standard of care for eligible patients within the therapeutic window of 0 to 4.5 hours from symptom onset. The goal of thrombolytic treatment is to restore blood flow to the affected brain tissue, thereby minimizing ischemic injury and improving functional outcomes.^[1,2]^ However, despite its proven efficacy in many cases, a significant proportion of patients undergoing thrombolysis experience poor prognoses, including persistent disability, neurological deterioration, or even death. Identifying and analyzing the risk factors for poor prognosis in these patients is essential to optimizing treatment strategies and improving patient outcomes.^[3–5]^

Several clinical, demographic, and imaging-related factors have been suggested to influence the prognosis of AIS patients treated with thrombolytics. Early detection of these factors may guide clinicians in identifying high-risk individuals who may benefit from more aggressive monitoring and individualized treatment plans. Risk factors such as age, comorbidities (e.g., diabetes, hypertension), baseline neurological status, infarct size, and the time-to-treatment interval are among the most frequently cited predictors of poor outcomes.^[6,7]^ Additionally, recent studies have highlighted the role of advanced neuroimaging techniques in assessing brain tissue salvageability and predicting functional recovery. The ischemic penumbra, a region of the brain that remains viable but at risk of irreversible damage, has emerged as a critical determinant in treatment success.^[8,9]^

In particular, demographic factors such as age and gender have shown consistent associations with stroke outcomes, with older age being a well-established risk factor for poor recovery. Comorbid conditions like atrial fibrillation (AF) and atherosclerosis have been linked to an increased risk of recurrent strokes, complicating the management of acute ischemic events. This article aims to identify and analyze the key risk factors associated with poor prognosis in patients with AIS undergoing thrombolytic therapy. By evaluating clinical parameters, this study seeks to contribute to the development of predictive models that can assist clinicians in making more informed decisions regarding treatment, patient monitoring, and long-term care strategies.

## 2. Methods

### 2.1. Study design

A retrospective evaluation was conducted at our hospital to identify and analyze the risk factors associated with poor prognosis in patients with AIS undergoing thrombolytic therapy. The study encompassed the period from January 2022 to August 2024. A total of 286 patients were included in the study, categorized into a favorable prognosis group (modified Rankin Scale (mRS) ≤ 2, n = 198) and an unfavorable prognosis group (mRS > 2, n = 88) based on their mRS scores 90 days post-treatment. The research methodology, intent, and protocols were developed in accordance with the Strengthening the Reporting of Observational Studies in Epidemiology (STROBE) guidelines.^[10]^ All subjects provided informed consent, and the study was rigorously reviewed by our hospital’s ethics committee. Adhering to relevant guidelines, our methods, design, performance, and reporting complied with the ethical standards of the Declaration of Helsinki for human research. Data confidentiality was maintained, with all personal identifiers removed to protect participant privacy.

### 2.2. Inclusion and exclusion criteria

Inclusion criteria comprised patients diagnosed with AIS based on clinical presentation and confirmed by neuroimaging (CT or MRI) within 24 hours of symptom onset, who received intravenous rt-PA therapy within the approved therapeutic window of 4.5 hours from symptom onset, provided informed consent, and maintained stable vital signs without significant systemic complications that would contraindicate thrombolytic therapy or participation in the study. Exclusion criteria included patients with non-ischemic stroke (such as hemorrhagic stroke, transient ischemic attack, or other non-ischemic neurological disorders like seizures, migraines, or brain tumors), those with absolute contraindications to rt-PA therapy (including recent major surgery within 14 days, active internal bleeding, history of intracranial hemorrhage, severe uncontrolled hypertension with systolic blood pressure > 185 mm Hg or diastolic blood pressure > 110 mm Hg, or known hypersensitivity to thrombolytic agents), patients with a baseline neurological impairments that would preclude meaningful assessment of recovery, and individuals with terminal illnesses or severe comorbid conditions that may compromise long-term survival and the ability to assess post-stroke recovery.

### 2.3. Data collection and outcome measures

Comprehensive clinical data were collected for all study participants, encompassing demographic characteristics and relevant medical history. Specifically, data included patient age, sex, history of smoking, and alcohol consumption. Additionally, information on underlying comorbidities such as hypertension, coronary artery disease, diabetes mellitus, and prior cerebral infarction was documented. Upon admission, each patient’s neurological status was assessed using the National Institutes of Health Stroke Scale (NIHSS). Furthermore, hematological parameters were recorded for all patients within 30 days post-admission to monitor any laboratory changes during the acute and subacute phases of treatment.

### 2.4. Statistical analysis

Statistical analyses were performed using SPSS version 26.0 (IBM Corp., Armonk) and R version 3.6.3 (R Foundation for Statistical Computing, Vienna, Austria). Continuous variables were expressed as mean ± standard deviation and compared between groups using independent samples *t* tests. Categorical variables were presented as frequencies and percentages, and comparisons between groups were conducted using chi-square tests, continuity-corrected chi-square tests, or Fisher exact tests as appropriate. Variables with a *P*-value <.10 in the univariate analysis were considered candidate predictors for inclusion in the multivariable logistic regression model. Subsequently, a forward stepwise selection approach was applied to build the final model, with entry and stay criteria set at *P* < .05 and *P* > .10, respectively. To identify risk factors for poor prognosis, multivariate logistic regression analysis with a forward selection method was employed, determining the odds ratios (OR) and their 95% confidence intervals (CI) for each significant variable. The rms package in R was utilized to develop a risk prediction nomogram and to generate receiver operating characteristic curves, facilitating the calculation of the concordance index (C-index) to assess the model’s discriminative ability. The optimal cutoff value for the prediction model was selected using the Youden index, allowing for the determination of sensitivity and specificity. Model calibration was evaluated using the Hosmer–Lemeshow goodness-of-fit test. Additionally, internal validation was performed through bootstrap resampling (n = 1000 iterations) to calculate the adjusted C-index and to construct calibration curves, which were visualized to assess the predictive performance of the nomogram. A *P*-value of <.05 was considered statistically significant. To address potential multicollinearity among predictors, particularly between leukocyte and neutrophil counts, we assessed collinearity before conducting multivariable logistic regression. Pearson correlation coefficients were calculated to examine pairwise correlations between variables, and variance inflation factors (VIF) were computed to quantify the degree of multicollinearity. A VIF threshold of < 5 was considered acceptable, with values < 2 indicating no meaningful collinearity.

## 3. Results

### 3.1. Clinical and laboratory differences between prognostic groups

Analysis of clinical and laboratory factors revealed significant differences between patients with good and poor prognosis following stroke. Patients in the poor prognosis group were more likely to be female and exhibited a higher prevalence of hypertension, diabetes, and a history of stroke or TIA. These findings emphasize the importance of underlying comorbidities and historical risk factors in determining patient outcomes. Neurological severity, assessed by NIHSS scores, was markedly higher in the poor prognosis group, indicating more severe neurological impairment at presentation. Laboratory parameters further highlighted significant disparities, with elevated levels of white blood cell (WBC), neutrophil count, D-dimer, and CRP observed in patients with poor prognosis. These findings suggest a potential link between systemic inflammation, coagulation activity, and adverse outcomes. Conversely, most routine hematological and biochemical markers, including lipid profiles (e.g., TC, LDL-C, HDL-C), coagulation indices (e.g., PT, APTT, FIB), and other hematologic parameters (e.g., PLT, MPV, MCH, MCHC), did not differ significantly between groups. Lifestyle factors such as smoking and drinking history also showed no notable impact on prognosis in this cohort. BMI did not differ significantly between groups (21.57 ± 3.67 vs 20.78 ± 3.12 kg/m²; *t* = 1.756, *P* = .081) (Table 1). These results underscore the multifactorial nature of stroke prognosis, with demographic factors, comorbid conditions, inflammatory markers, and neurological severity playing pivotal roles.

**Table 1:** Comparison of clinical factors between good and poor prognosis groups in patients with stroke or TIA.

Factors	Good prognosis (n = 198)	Poor prognosis (n = 88)	*χ*²/*t* value	*P*-value
Gender (male/female)	137 (69.19%)/61 (30.81%)	41 (46.59%)/47 (53.41%)	13.24	<.001
Body mass index	21.57 ± 3.67	20.78 ± 3.12	1.756	.081
Hypertension	90 (45.45%)	61 (69.32%)	13.92	<.001
Diabetes	31 (15.65%)	45 (51.14%)	39.3	<.001
Stroke/TIA history	22 (11.11%)	29 (33.95%)	19.84	<.001
NIHSS score (points)	2.94 ± 2.44	9.11 ± 7.70	10.20	<.001
WBC (×10^9^/L)	7.28 ± 2.40	11.85 ± 3.08	13.58	<.001
Neu count (×10^9^/L)	5.23 ± 2.27	7.96 ± 2.51	9.082	<.001
D-dimer (mg/L)	1.34 ± 1.36	3.51 ± 1.99	10.72	<.001
PDW (%)	15.91 ± 1.08	15.92 ± 0.36	0.03	.975
TC (mmol/L)	6.74 ± 1.76	6.33 ± 1.21	0.09	.931
MCHC (g/L)	350.50 ± 9.81	346.42 ± 8.73	0.098	.925
Smoking history	58 (29.29%)	25 (28.41%)	0.019	.889
PLT (×10^9^/L)	217.26 ± 70.45	220.00 ± 76.93	0.218	.835
LDL-C (mmol/L)	2.80 ± 0.94	2.90 ± 0.80	0.227	.824
TT (s)	13.61 ± 1.65	14.01 ± 1.41	0.275	.777
Coronary disease	36 (18.18%)	14 (15.91%)	0.169	.681
P-LCR (%)	24.63 ± 8.38	24.04 ± 8.87	0.462	.656
PT (s)	11.15 ± 0.87	11.26 ± 0.56	0.449	.652
TP (g/L)	66.50 ± 6.71	64.65 ± 6.48	0.456	.647
APTT (s)	32.71 ± 3.14	30.00 ± 3.29	0.537	.574
TG (mmol/L)	1.59 ± 1.15	1.39 ± 0.69	0.633	.546
MPV (fL)	9.33 ± 1.23	9.94 ± 1.33	0.612	.531
HGB (g/L)	140.16 ± 14.33	141.70 ± 15.75	0.659	.526
PCT (%)	0.22 ± 0.06	0.21 ± 0.07	0.618	.522
Drinking history	72 (36.36%)	27 (30.68%)	0.43	.512
P-LCC (%)	47.68 ± 19.92	48.77 ± 17.55	0.776	.435
INR	1.01 ± 0.08	1.01 ± 0.05	0.796	.435
HCT (%)	41.72 ± 4.07	42.81 ± 4.62	0.767	.432
FIB (g/L)	2.97 ± 0.59	3.05 ± 0.62	0.335	.403
HDL-C (mmol/L)	1.15 ± 0.30	1.23 ± 0.29	0.882	.397
ALB (g/L)	36.28 ± 5.08	35.18 ± 3.02	0.896	.376
Hcy (µmol/L)	14.82 ± 11.83	18.60 ± 11.27	1.143	.256
Age (years)	65.41 ± 11.39	67.13 ± 10.26	1.214	.226
RBC (×10¹²/L)	4.38 ± 0.52	4.73 ± 0.59	5.037	.222
MCV (fL)	97.18 ± 4.78	90.77 ± 5.21	1.356	.192
MCH (pg)	32.22 ± 2.08	32.16 ± 1.69	1.367	.192
RDW (fL)	12.76 ± 0.78	13.53 ± 0.76	1.385	.156
GLO (g/L)	26.23 ± 5.31	27.10 ± 2.66	1.456	.153
CRP (mg/L)	3.93 ± 3.39	8.87 ± 8.25	7.182	.001

Age = age, ALB = albumin, APTT = activated partial thromboplastin time, CRP = C-reactive protein, FIB = fibrinogen, GLO = globulin, HCT = hematocrit, Hcy = homocysteine, HDL-C = high-density lipoprotein cholesterol, HGB = hemoglobin, INR = International Normalized Ratio, LDL-C = low-density lipoprotein cholesterol, MCH = mean corpuscular hemoglobin, MCHC = mean corpuscular hemoglobin concentration, MCV = mean corpuscular volume, MPV = mean platelet volume, NIHSS = National Institutes of Health Stroke Scale Score, PCT = platelet crit, PDW = platelet distribution width, P-LCC = platelet large cell concentration, P-LCR = platelet large cell ratio, PLT = platelet count, PT = prothrombin time, RBC = red blood cell count, RDW = red cell distribution width, TC = total cholesterol, TG = triglycerides, TIA = transient ischemic attack, TP = total protein, TT = thrombin time, WBC = white blood cell count.

### 3.2. Multivariate logistic regression analysis of prognostic factors

Multivariate logistic regression analysis identified several independent predictors of poor prognosis in patients with stroke. Among clinical and laboratory factors, diabetes emerged as a significant risk factor, with an OR of 6.511 (95% CI: 1.667–24.605, *P* = .008), indicating that patients with diabetes were more than 6 times as likely to have a poor prognosis. Neurological severity at admission, assessed using NIHSS scores, was also a robust predictor of outcomes. Each unit increase in NIHSS score was associated with a 35.4% higher likelihood of poor prognosis (OR: 1.354, 95% CI: 1.097–1.565, *P* = .001). Inflammatory and hematological markers played a critical role in predicting prognosis. Elevated WBC counts (OR: 1.459, 95% CI: 1.114–1.786, *P* = .002) and neutrophil counts (OR: 1.402, 95% CI: 1.037–1.758, *P* = .022) were significantly associated with increased risk of poor outcomes. Additionally, D-dimer, a marker of coagulation and fibrinolysis activity, was strongly associated with prognosis, with an OR of 2.088 (95% CI: 1.360–2.988, *P* = .001), emphasizing its relevance as a prognostic biomarker. Other factors, such as gender, hypertension, CRP levels, and history of stroke or TIA, did not reach statistical significance in the multivariate model, suggesting that their contributions to prognosis may be less pronounced when considered alongside the more dominant predictors (Table 2). Collinearity diagnostics demonstrated that leukocyte and neutrophil counts were moderately correlated (Pearson *R* = 0.68). However, both variables exhibited low VIF values (WBC: 1.89; neutrophil count: 1.92), well below the conventional threshold of concern (VIF < 5). These results indicate no significant multicollinearity. Consistent with this, the multivariable logistic regression model retained both leukocyte count (OR = 1.459, 95% CI: 1.114–1.786, *P* = .002) and neutrophil count (OR = 1.402, 95% CI: 1.037–1.758, *P* = .022) as independent predictors of poor prognosis.

**Table 2:** Multivariate logistic regression analysis of clinical factors.

Factors	β-value	Standard error value	Wald value	OR value	95% CI for OR	*P*-values
Diabetes	1.804	0.665	7.005	6.511	1.667–24.605	.008
Admission NIHSS score	0.289	0.091	11.482	1.354	1.097–1.565	.001
WBC	0.365	0.124	8.958	1.459	1.114–1.786	.002
Neu count	0.328	0.144	4.945	1.402	1.037–1.758	.022
D-dimer	0.708	0.197	11.373	2.088	1.360–2.988	.001
Gender	0.050	0.120	0.170	1.050	0.830–1.330	.681
Hypertension	0.150	0.160	0.880	1.160	0.850–1.590	.356
CRP	0.250	0.130	3.700	1.280	0.986–1.660	.068
Stroke/TIA history	0.220	0.140	2.460	1.250	0.950–1.640	.095

CI = confidence interval, CRP = C-reactive protein, Neu count = neutrophil count, NIHSS score = National Institutes of Health Stroke Scale score, OR = odds ratio, SE = standard error, TIA = transient ischemic attack, WBC = white blood cell count, β = regression coefficient.

### 3.3. Development of a nomogram prediction model for poor prognosis

**Figure 1.**
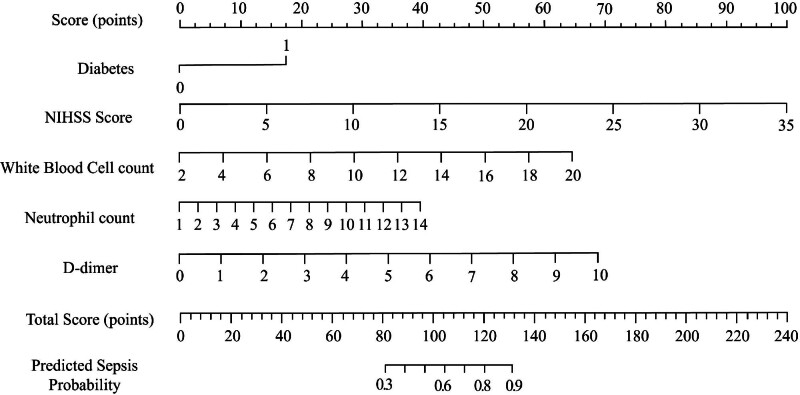
Nomogram predicting poor prognosis in patients with acute ischemic stroke treated with thrombolytic therapy.

Based on the results of multivariate logistic regression analysis, 5 independent predictors of poor prognosis following thrombolytic therapy in AIS patients were identified: diabetes history, NIHSS score, WBC count, neutrophil count, and D-dimer levels. These variables were used to construct a nomogram prediction model to estimate the probability of poor outcomes. The nomogram assigns a specific score to each predictor, allowing the total score for an individual patient to be calculated by summing the scores of all included factors. The total score corresponds to a probability value on the bottom axis of the nomogram, representing the estimated risk of poor prognosis. Higher total scores indicate an increased likelihood of adverse outcomes, emphasizing the additive effect of multiple risk factors on prognosis (Fig. 1).

### 3.4. Discriminative ability and validation of the nomogram prediction model

**Figure 2.**
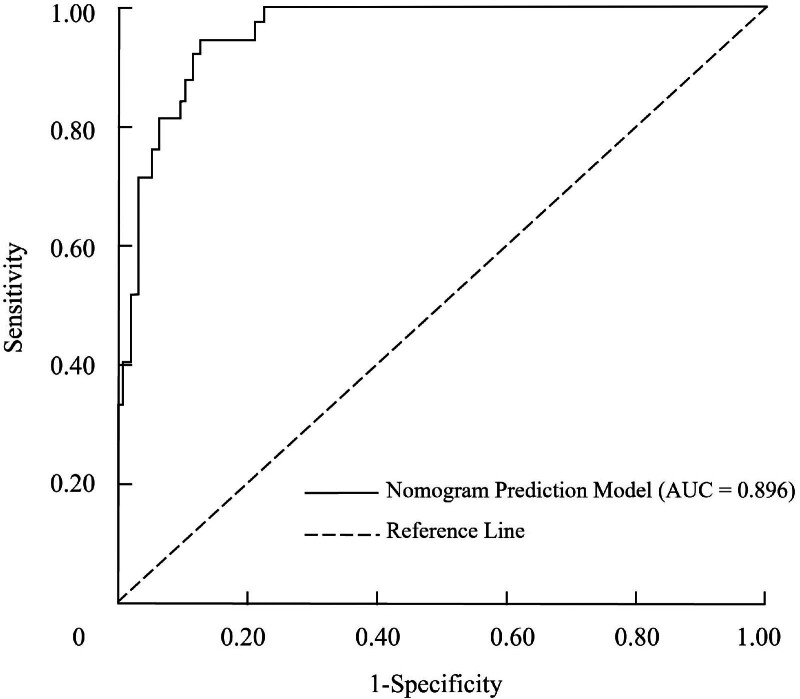
Receiver operating characteristic (ROC) curve demonstrating the discriminative ability of the nomogram prediction model for poor prognosis in acute ischemic stroke.

The predictive performance of the nomogram for poor prognosis in AIS patients following thrombolytic therapy was evaluated using receiver operating characteristic analysis. The AUC for the model was 0.896 (95% CI: 0.781–0.958), demonstrating excellent discriminative ability. When the cutoff value was set to maximize the Youden index, the model achieved a sensitivity of 88.1% and a specificity of 82.9%, indicating strong capability to distinguish between patients with good and poor prognoses. To ensure the robustness of the model, internal validation was conducted using Bootstrap resampling, with 1000 iterations. The adjusted C-index was 0.728, reflecting the model’s reliable predictive accuracy. Calibration was further assessed by plotting a calibration curve, which revealed a mean absolute error of 0.008 between predicted and observed probabilities, suggesting strong agreement between predicted risk and actual outcomes (Fig. 2).

### 3.5. Calibration of the nomogram prediction model

**Figure 3.**
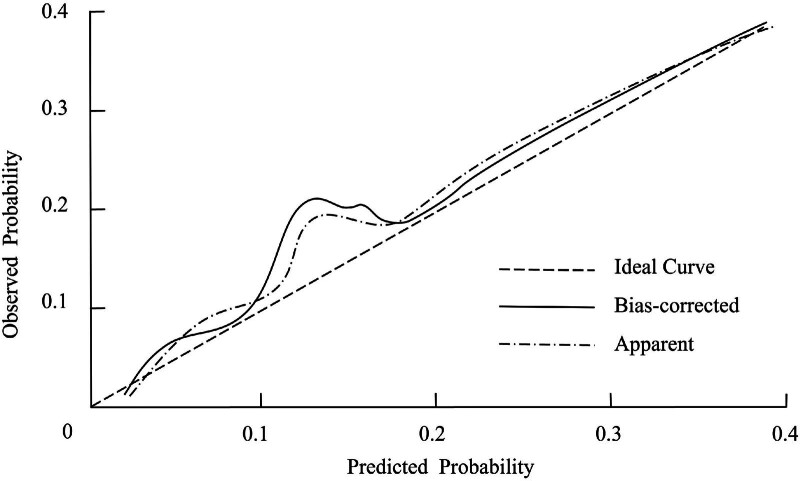
Calibration curve assessing the agreement between predicted and observed probabilities in the nomogram prediction model for poor prognosis in acute ischemic stroke.

The calibration of the nomogram prediction model for poor prognosis in AIS patients following thrombolytic therapy was assessed using the Hosmer–Lemeshow goodness-of-fit test. The test result yielded a *χ*² value of 2.916 with a *P*-value of .856, indicating that the model’s predictions were well-calibrated and consistent with observed outcomes. The calibration curve further supported the model’s predictive reliability, demonstrating strong alignment between the predicted and observed probabilities. Both the predicted and observed calibration lines showed excellent agreement, closely overlapping the diagonal line representing perfect calibration. This indicates that the model is capable of accurately estimating the risk of poor prognosis across different probability levels (Fig. 3).

### 3.6. Clinical utility of the nomogram prediction model

**Figure 4.**
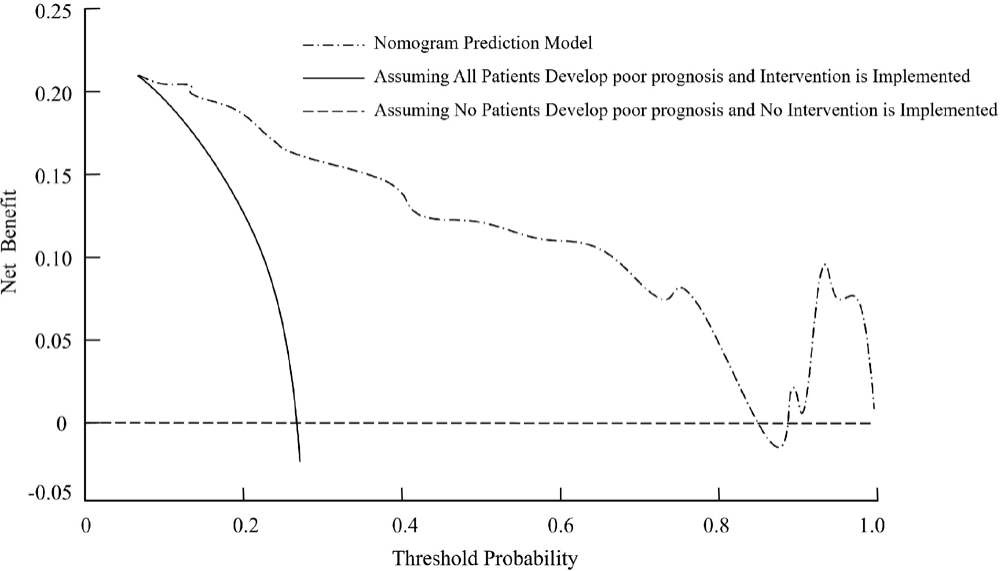
Decision curve analysis (DCA) illustrating the clinical utility of the nomogram prediction model for poor prognosis in acute ischemic stroke patients.

The clinical effectiveness of the nomogram prediction model was evaluated using decision curve analysis (DCA). In the DCA plot, the horizontal line represents the assumption that no patients experience poor prognosis and thus no intervention is provided, yielding a net benefit of 0. Conversely, the diagonal line represents the assumption that all patients are at risk of poor prognosis and receive interventions, resulting in a negative net benefit due to unnecessary interventions for low-risk individuals. The nomogram prediction model demonstrated significantly higher net benefit compared to both extreme scenarios across a range of threshold probabilities. Specifically, DCA showed that the model’s net benefit exceeds that of the “treat-none” and “treat-all” strategies when the threshold probability for poor prognosis lies approximately between 0.15 and 0.60. Within this interval, clinicians can use the predicted risk to guide interventions: for example, if an individual patient’s calculated probability of poor 90-day outcome exceeds 0.25, intensified management (such as early transfer to a stroke unit with continuous hemodynamic monitoring, more aggressive antithrombotic adjustment, or targeted nursing resources) may be warranted. At lower thresholds (<0.15), the net benefit of pursuing additional interventions is marginal or negative relative to the “treat-none” strategy, indicating that routine care may suffice. Conversely, at very high thresholds (>0.60), the incremental benefit over treating all patients becomes negligible, since almost all high-risk individuals are already captured. In clinical practice, selecting a cutoff around 0.25 may be reasonable: patients with a predicted risk above this level could receive intensified monitoring or tailored interventions, whereas those below could continue standard care. This threshold balances the avoidance of unnecessary treatments in low-risk individuals with the timely identification of high‐risk patients (Fig. 4).

### 3.7. Post hoc power analysis

To assess the adequacy of our sample size for logistic regression with 5 independent predictors (diabetes, NIHSS score, WBC count, neutrophil count, and D-dimer), we conducted a post hoc power analysis using the observed differences between the good and poor prognosis groups. For categorical variables (diabetes), Cohen h was calculated based on the observed proportions; for continuous variables (NIHSS, WBC count, neutrophil count, and D-dimer), Cohen *d* was computed using group means and standard deviations. The resulting effect sizes ranged from 0.78 to 1.65, corresponding to post hoc statistical power values exceeding 0.99 (α = 0.05, 2-sided). These results confirm that our sample size (n = 286; 88 events) was sufficient to detect the observed effects with high statistical power.

## 4. Discussion

This study identified and analyzed key clinical and laboratory factors influencing the prognosis of patients with AIS undergoing thrombolytic therapy. The results underscore the multifactorial nature of stroke prognosis, with significant contributions from demographic, clinical, and laboratory markers. The development and validation of a nomogram prediction model further highlight the potential for individualized risk stratification to improve clinical decision-making and patient outcomes. The nomogram developed in this study integrates 5 independent predictors, diabetes, NIHSS score, WBC count, neutrophil count, and D-dimer levels, to estimate the probability of poor prognosis. The high AUC value of 0.896 demonstrates excellent discriminative ability, while robust calibration and validation results confirm its reliability in clinical settings. The nomogram allows for individualized risk assessment by assigning a cumulative score based on patient-specific variables. This tool provides a practical and accessible method for clinicians to stratify patients according to their risk and tailor interventions accordingly. The clinical utility of the prediction model was further demonstrated by decision curve analysis, which revealed significantly higher net benefit across a range of threshold probabilities compared to extreme intervention scenarios. By identifying patients at high risk of poor outcomes, the model supports targeted clinical interventions, such as more aggressive pharmacological treatment, specialized nursing care, or enhanced monitoring.^[11,12]^ This approach optimizes resource allocation, reduces unnecessary treatments in low-risk patients, and improves overall care efficiency.^[13,14]^

Among the independent predictors, diabetes emerged as one of the most significant risk factors, with an odds ratio exceeding 6. The association between diabetes and poor stroke prognosis may be explained by the chronic vascular and metabolic changes induced by diabetes, including endothelial dysfunction, accelerated atherosclerosis, and pro-inflammatory states. These changes contribute to reduced cerebral perfusion, impaired vascular repair, and increased susceptibility to thromboembolic events, complicating the recovery process after stroke.^[15,16]^ The NIHSS score, a widely recognized measure of neurological severity, was another robust predictor of poor outcomes. Higher NIHSS scores reflect greater initial neurological impairment, which is often linked to larger infarct size, extensive brain tissue damage, and reduced potential for functional recovery. This finding reinforces the critical need for rapid diagnosis and treatment to minimize the extent of ischemic injury and improve long-term outcomes.^[17,18]^

Elevated levels of inflammatory markers, including WBC and neutrophil counts, were significantly associated with poor prognosis. Systemic inflammation plays a pivotal role in ischemic stroke by exacerbating neuronal damage, increasing blood–brain barrier permeability, and promoting secondary injury through pro-inflammatory cytokines and reactive oxygen species. Elevated WBC and neutrophil counts serve as indicators of systemic inflammation and may signify heightened immune response contributing to adverse outcomes. D-dimer, a marker of coagulation and fibrinolysis, was another strong predictor of poor prognosis.^[19]^ Elevated D-dimer levels reflect increased thrombus burden and ongoing fibrinolysis, which may indicate a hypercoagulable state. This is particularly relevant in stroke patients, as impaired clot resolution and microvascular obstruction can exacerbate ischemic injury, delay reperfusion, and hinder recovery. These findings highlight the importance of addressing coagulation abnormalities in the acute management of stroke patients.

In our cohort, the prevalence of poor 90-day outcome was 30.8% (88/286). The DCA curve demonstrated its greatest net benefit within the 0.20 to 0.30 region; therefore, we designated 0.25 as a practical operating point. Decision-analytically, a threshold probability *P*_*t*_ = .25 implies a false-positive penalty of *P*_*t*_/(1 - *P*_*t*_) = .33, that is, 1 false positive is considered one-third as harmful as a missed true positive. Given that the actions triggered above this threshold (enhanced monitoring in a stroke unit, earlier tailored rehabilitation, and intensified secondary prevention) are of moderate burden and low procedural risk, this exchange rate is clinically reasonable. Consistent with this rationale, net benefit at 0.25 was high and comparable to neighboring cutoffs (0.20–0.30), while thresholds < 0.15 favored overtreatment and those > 0.60 approximated a “treat-all” strategy. Accordingly, using 0.25 as an operational cutoff balances resource use and risk mitigation in routine practice.

Traditional predictors such as older age, AF, and hypertension did not remain significant in our multivariable model, which contrasts with prior reports. Several factors likely explain this discrepancy. First, our cohort comprised rt-PA-treated AIS patients within 4.5 hours, managed under standardized pathways. In this acute, protocolized context, the prognostic impact of baseline clinical risks may be partially mediated by neurological severity and acute pathobiology. After adjusting for NIHSS and inflammatory/coagulation markers, WBC, neutrophil count, and D-dimer, the direct associations of age and hypertension were attenuated, consistent with mediation or overadjustment. Second, risk factor ascertainment reflected admission status rather than longitudinal exposure. A single blood pressure measurement or a history label may not capture blood pressure variability, treatment responsiveness, or chronic burden; similarly, AF around the index event may be under-recognized or misclassified, biasing effects toward the null. Third, sample characteristics and power considerations matter. Age distribution was relatively homogeneous, and AF prevalence was low, limiting variation to detect modest effects. Although the events-per-variable ratio was acceptable for the retained predictors (88 events; ≈17.6 EPV for 5 covariates), the study was underpowered to detect small to moderate odds ratios for traditional risks once stronger, proximal predictors were included. Fourth, modeling choices may have contributed. We used stepwise selection with prespecified entry/removal thresholds; this can exclude clinically relevant but statistically borderline variables and does not account for potential non-linearities or interactions. These findings underscore that, in thrombolysed AIS, proximal severity and acute inflammatory–coagulatory activity can dominate risk stratification at 90 days.^[20,21]^ To address residual uncertainty, we plan external, multicenter validation, sensitivity analyses forcing age, hypertension, and AF into the model, assessment of non-linearity via restricted cubic splines, exploration of interactions (e.g., age × NIHSS), and penalized regression to reduce selection bias.^[22–24]^ Such work will clarify whether traditional risks add incremental value beyond acute severity and biology in this treatment setting.

A major strength of this study is the comprehensive analysis of both clinical and laboratory factors, allowing for a holistic understanding of stroke prognosis. The use of internal validation and calibration further enhances the robustness of the model. This study has several limitations. First, the retrospective and single-center design may introduce selection bias and limit the generalizability of the findings. Although the sample size (n = 286) was statistically adequate for model development, as supported by post hoc power analysis with high power for all retained predictors, the external validity of the model remains uncertain, particularly in more diverse clinical populations. Second, variable selection was based on univariate screening (*P* < .10) followed by forward stepwise logistic regression. While this approach improves model parsimony, it may exclude clinically important but statistically borderline variables, such as hypertension, CRP, and prior stroke or TIA. However, these variables did not show independent predictive value after adjusting for stronger covariates, and collinearity diagnostics confirmed no substantial multicollinearity among the included predictors. Third, although internal validation via bootstrap resampling (1000 iterations) was performed to mitigate optimism bias, the reduction from the original AUC (0.896) to the bootstrap-corrected C-index (0.728) suggests some degree of overfitting. Additionally, the model lacks external validation, limiting its transportability and applicability in broader practice. To enhance robustness and clinical utility, future research should involve prospective, multicenter cohorts with heterogeneous populations to externally validate the nomogram. Further analyses should explore potential non-linear relationships and interactions (e.g., age by NIHSS), and incorporate penalized regression techniques to reduce overfitting and improve generalizability. These steps are essential for confirming the model’s stability and supporting its integration into individualized stroke management strategies.

## 5. Conclusions

This retrospective study suggests that diabetes, NIHSS score, WBC count, neutrophil count, and D-dimer levels may serve as potential predictors of poor prognosis in patients with AIS undergoing thrombolytic therapy. The nomogram developed based on these factors demonstrated promising discriminative performance, calibration, and clinical utility in internal validation. These findings indicate that integrating clinical severity and laboratory biomarkers could support individualized risk assessment and inform prognosis-oriented decision-making. However, the model’s applicability requires further validation in prospective, multicenter cohorts before it can be widely implemented in clinical practice.

## Acknowledgments

We appreciate the contributions of all the participants.

## Author contributions

**Conceptualization:** Cuihong Ma, Lixia Gao, Lei Chen.

**Data curation:** Cuihong Ma, Lixia Gao.

**Formal analysis:** Cuihong Ma, Lixia Gao, Jing Xu.

**Investigation:** Cuihong Ma, Lixia Gao, Zengkun Hong, Jing Xu.

**Methodology:** Cuihong Ma, Lixia Gao, Zengkun Hong, Jing Xu, Lei Chen.

**Resources:** Cuihong Ma, Zengkun Hong, Jing Xu.

**Software:** Cuihong Ma, Zengkun Hong.

**Writing – original draft:** Cuihong Ma, Lixia Gao.

**Writing – review & editing:** Lei Chen.
